# A two-layered mechanical model of the rat esophagus. Experiment and theory

**DOI:** 10.1186/1475-925X-3-40

**Published:** 2004-11-01

**Authors:** Yanhua Fan, Hans Gregersen, Ghassan S Kassab

**Affiliations:** 1 Institute of Experimental Clinical Research, Skejby Hospital, Aarhus, Denmark; 2 Center for Biomechanics and Visceral Pain, Aalborg Hospital, Aalborg, Denmark; 3 Center for Sensory-Motor Interaction, Aalborg University, Aalborg, Denmark; 4 Haukeland University Hospital, Bergen, Norway; 5 Department of Biomedical Engineering, UC Irvine, Irvine, California

**Keywords:** Biomechanics, Mucosa-submucosa layer, Muscle layer, Opening angle, Zero-stress state

## Abstract

**Background:**

The function of esophagus is to move food by peristaltic motion which is the result of the interaction of the tissue forces in the esophageal wall and the hydrodynamic forces in the food bolus. The structure of the esophagus is layered. In this paper, the esophagus is treated as a two-layered structure consisting of an inner collagen-rich submucosa layer and an outer muscle layer. We developed a model and experimental setup for determination of elastic moduli in the two layers in circumferential direction and related the measured elastic modulus of the intact esophagus to the elastic modulus computed from the elastic moduli of the two layers.

**Methods:**

Inflation experiments were done at *in vivo *length and pressure-diameters relations were recorded for the rat esophagus. Furthermore, the zero-stress state was taken into consideration.

**Results:**

The radius and the strain increased as function of pressure in the intact as well as in the individual layers of the esophagus. At pressures higher than 1.5 cmH_2_O the muscle layer had a larger radius and strain than the mucosa-submucosa layer. The strain for the intact esophagus and for the muscle layer was negative at low pressures indicating the presence of residual strains in the tissue. The stress-strain curve for the submucosa-mucosa layer was shifted to the left of the curves for the muscle layer and for the intact esophagus at strains higher than 0.3. The tangent modulus was highest in the submucosa-mucosa layer, indicating that the submucosa-mucosa has the highest stiffness. A good agreement was found between the measured elastic modulus of the intact esophagus and the elastic modulus computed from the elastic moduli of the two separated layers.

## Introduction

The majority of previous mechanical studies on visceral organs, including the blood vessels, have considered them as homogenous tubes; i.e., a single layer structure. Most visceral organs are, however, multilayered, e.g. the arteries consist of intima, media and adventitia and the gastrointestinal tract has circumferential and longitudinal muscle layers, submucosa and mucosa layers.

The esophagus represents a very interesting biomechanical model since it is the only organ that can be separated into two layers without damage to either layer. Hence, the muscle layers can be separated from the mucosa-submucosa layer by dissection, leaving two intact tubes. Separation experiments of the esophagus in guinea pigs and rabbits showed that the submucosa-mucosa layer had larger residual strains and opening angles than the muscle layer [1-3]. Considering the multi-layered composite structure and the difference in zero-stress state between the layers, the stress distribution in the wall is expected to be non-homogeneous. Hence, the material constants likely differ between the layers. Such a finding impacts our understanding of biological tissue remodelling and the function of mechanosensitive receptors located in various layers of the wall [4-6]. Therefore, data on the strain and stress distribution in the layers will facilitate the understanding of the relationship between the stress, remodelling of the tissue and sensory responses. To pursue this line of study, however, it is necessary to know how the stress and strain in the esophagus can be computed for each layer, and how the composite can be put together to give the overall observed mechanical properties.

In this study we recognize that the esophagus consists of mucosa-submucosa and muscle layers. We analyze these layers as elastic shells. Each layer has its own zero-stress state, and its own elastic constants. We will determine the material properties of each layer separately. Specifically, the material properties in the individual layers will be computed from the pressure-diameter relation and zero-stress state with the method of analysis presented below. We will then propose a simple model to combine the two layers to predict the overall behavior of the esophagus under certain hypotheses. The limitations and implications of the model will be discussed.

## Materials and methods

Eight male Wistar rats, weighing 380–420 grams, were used in the study. Approval of the protocol was obtained from the Danish Animal Experiment Committee. The animals were anesthetized with sodium pentobarbital (50 mg kg^-1 ^ip). Papaverine (15 mg) was injected into the tail vein to relax the visceral muscles and to euthanize the rat. The cervical segment of the esophagus was dissected free from its adjacent tissue. Next, the thoracic and abdominal cavities were opened. After pouring cold Krebs solution into the thoracic cavity, the esophagus was quickly dissected free from adjacent tissues and its *in situ *length was measured. A 2-cm-long segment from the middle part, intended for the distension test, was marked. The length of this segment and that of the entire esophagus was measured. The entire esophagus was then cut at the proximal and distal ends including the very first part of the stomach, and immediately placed in calcium-free Krebs solution containing 6% dextran and 0.25% ethylene glycol-bis (β-aminoethyl ether)-N,N,N,N-tetraacetic acid (EGTA). The solution was aerated with a gas mixture of 95% O_2_-5% CO_2 _at pH of 7.4. After careful removal of all extra-esophageal tissue, the length was measured *in vitro*.

### Pressure-diameter experiments

The middle part of the intact esophagus was mounted in an organ bath containing the Ca^+2^-free Krebs solution. The segment was stretched to the *in vivo *length and fixed. The distal end was closed whereas the proximal end was cannulated and connected to a fluid container. After preconditioning the tissue with pressures up to 8 cmH_2_O, a ramp test was performed where the pressure was changed continuously at a rate of 2 cmH_2_O per minute up to a pressure of 8 cmH_2_O. A video camera (Sony CCD camera) monitored the changes in diameter and length during the distension and images were grabbed by a PC.

After the test of the intact esophagus, the muscle and submucosa-mucosa layers were gently separated into two tubes. The tubes were studied separately using the same procedure outlined above. The only difference was that the maximum pressure was set to 6 cmH_2_O.

### The zero-stress state of the esophagus

The zero-stress state of the esophagus was obtained in accordance with the method used for blood vessels [7]. Briefly, six rings of 1 mm length were cut from the intact esophagus and from the separated layers and were then cut in the radial direction to obtain the zero-stress state. The choice of the ring length was based on pilot studies. The radial cut caused the rings to open up into sectors. The shape of each ring segment at the zero-stress state was photographed 60 minutes after the radial cut to allow the creep to subside.

### Data analysis

The morphometric measurements were made using SigmaScan Pro image analysis software (Jandel Scientific, Germany). The data were obtained from the images of the tubes in the distended state, rings in the no-load state and sectors in the zero-stress state. In the distended state for both the intact esophagus and the separated tubes, images were analyzed for each 0.5 cmH_2_O increment. The outer diameter was measured at each pressure level and averaged over three locations. At the no-load and zero-stress states, the inner and outer circumferential lengths were measured along with the thickness and area of the wall and layers (for calculation of inner, outer or mid-layer circumference). The opening angle was defined as the angle subtended between two radii drawn from the midpoint of the inner wall to the tips of the inner wall of the open sector.

The stresses and strains of the esophagus and its sublayers in the pressurized state were determined under the assumption that the geometric configuration of the lumen is cylindrical, the wall of the esophagus is incompressible, and the material in each layer is homogenous. Based on the above measurements and assumptions, parameters of the esophagus such as the luminal radius (r_i-p_), the wall thickness (H_p_), the mid-wall circumference (C_m-p_) at a given pressure were computed as r_i-p _= [(r^2^_o-p _- A_n_/*πλ*_1_)]^1/2^, H_p _= r_o-p _- r_i-p_, and C_m-p _= 2*π*(r_i-p _+ H_p_/2). The outer radius (r_o-p_) of the intestine was computed according to the outer diameter (D_o_). The circumferential Green's strains and Kirchhoff's stress were computed according to the equations:



where Cm-z is the mid-wall circumference at the zero-stress state.



where 

The tangent modulus can be estimated from the slope of the stress-strain relation as



The tangent modulus given by Eq. (3) corresponds to Young's modulus in the linear stress-strain regime.

### Integration of Two-Layers: An Analytical Model

We assume that the circumferential stress-strain relationships for the inflation experiment obey Hooke's law for each layer

*σ*_*θθ*_^(*sm*) ^= *E*_*θθ*_^(*sm*)^*e*_*θθ*_^(*sm*) ^*σ*_*θθ*_^(*m*) ^= *E*_*θθ*_^(*m*)^*e*_*θθ*_^(*m*) ^    (4)

where *σ*, *E *and *e *indicate the Cauchy stress, Tangent modulus and Green strain, respectively. *θθ*, sm and m indicate the circumferential direction and the submucosal and muscle layers, respectively.

In general, the circumferential stress is a function of circumferential and longitudinal strain. In the present analysis, we assume that the cross-modulus is small such that the longitudinal term is negligible in comparison with the circumferential term and hence eq. (4). We further assume that the esophagus is a circular cylinder. The basic equations of equilibrium and deformation are given in Flugge [8]. Let x denote the longitudinal axis, *θ *the circumferential axis and z the radial coordinate. N_*θ *_denotes the tensile membrane stress resultant in *θ *direction. The displacement in the x, *θ*, and z directions of a point on the neutral axis surface are denoted by u, v, and w, respectively. The displacements of any point, A, is denoted by u_A_, v_A_, w_A _as follows: 1) u_A _= displacement along the generator, positive in the direction of increasing x; 2) v_A _= displacement along a circle of radius a + z, positive in the direction of increasing *θ *and 3) w_*A *_= radial displacement, positive outward.

According to the Bernoulli-Kirchhoff hypothesis (all points lying on one normal to the neutral surface before deformation remain on the normal after deformation), we have



where a indicate the neutral axis.

The circumferential strain which is assumed to be small is given by



The circumferential membrane stress resultant N_*θ *_is given by



By substitution of Eqs. (4), (5), and (6) into Eq. (7) and noting that in the inflation experiments u, v, and w do not change with *θ *and w does not change with x, we obtain



Eq. (8) can be integrated to yield



where I stands for intact esophagus; a^I^, a^sm ^and a^m ^are the neutral axes for the submucosa (1.35) and muscle (1.15). Equation (9) can be solved in terms of E^I ^as



Hence, we can compare measured E^I ^from Eq. (3) with E^I ^computed from E^sm ^and E^m ^as given by Eq. (9b).

### Statistical Analysis

The data were assumed to be representative of a normal distribution. The results are expressed as means ± SE. Student's t test and analysis of variance were used to detect possible differences between curves obtained from the intact esophagus and the two sublayers. The results were regarded as significant if P < 0.05.

## Results

The esophagi shortened by approximately 30% after excision. However, the segments were stretched to the *in vivo *length before the mechanical tests. The length was fixed during the distension protocol.

**Figure 1:**
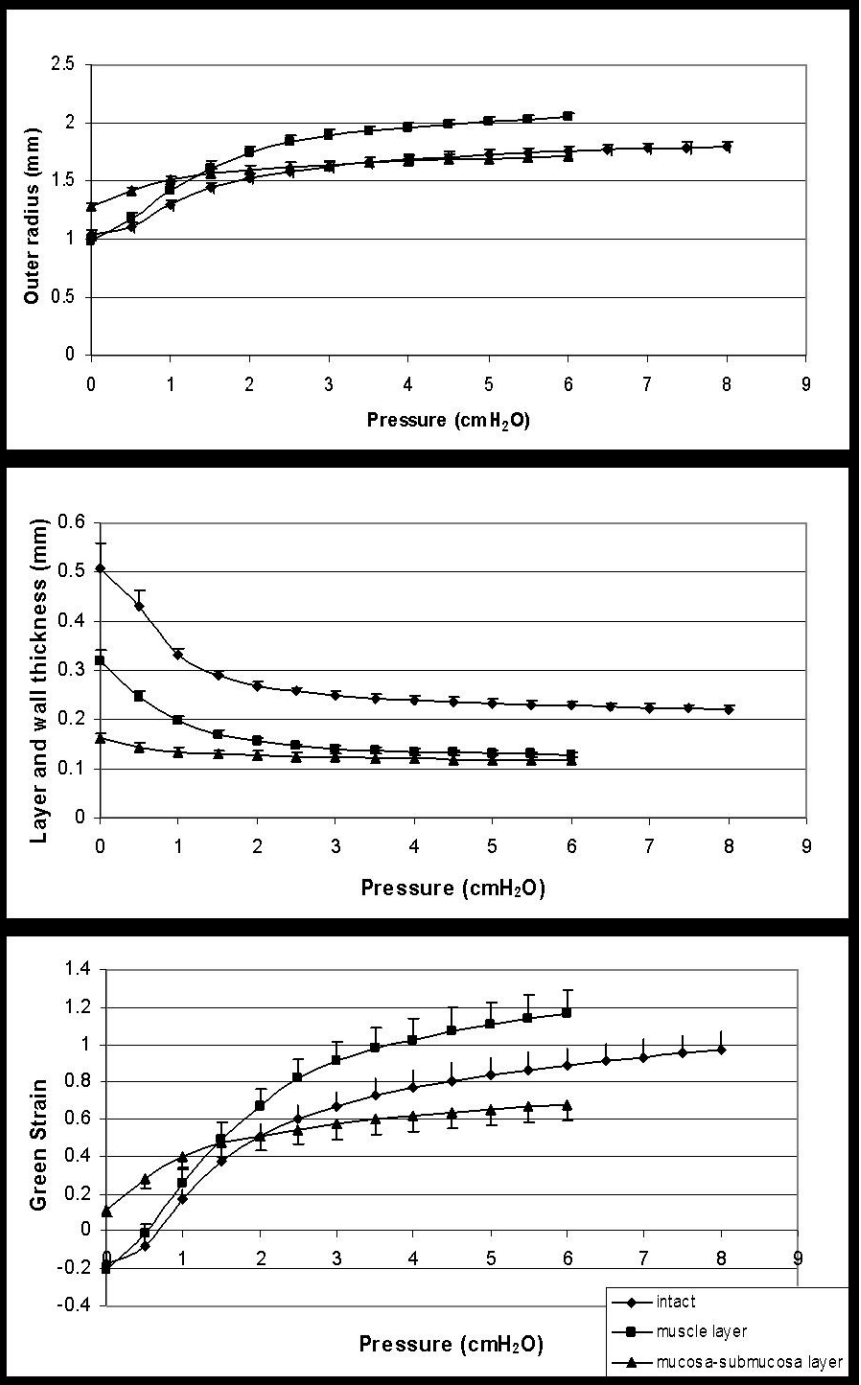
Outer radius (top graph), wall thickness (middle graph) and circumferential Green's strain in the intact esophagus and the muscle and mucosa-submucosa sublayers as function of pressure. Values are means ± SE.

The outer radius, wall thickness, and circumferential Green's strain as function of pressure is shown in figure 1. The radius and the Green's strain increased as function of pressure in the intact as well as the separated esophagus. At pressures higher than 1.5 cmH_2_O the muscle layer had a higher radius and strain than the mucosa-submucosa layer. The strain for the intact esophagus and for the muscle layer was negative at low pressures indicating the presence of residual strains in the tissue. The thickness of the intact wall and the separated layers decreased as function of pressure. The mucosa-submucosa was the thinnest layer.

**Figure 2:**
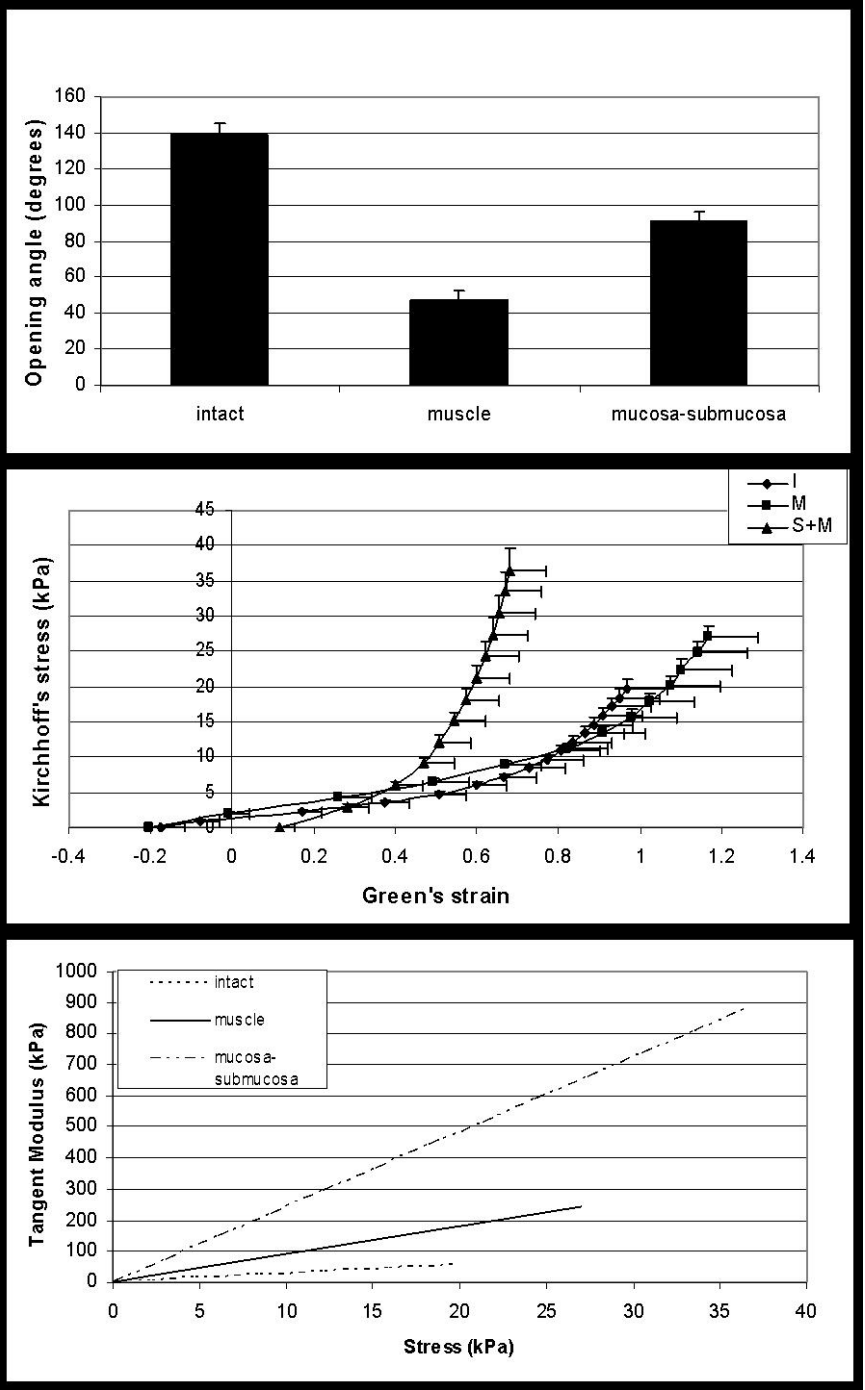
Opening angle after anterior cut in the intact esophagus and after separation into the muscle and mucosa-submucosa layers (top) and the stress-strain relationship in sense of Kirchhoff and Green (middle). Values are means ± SE from 8 rats. The bottom graph shows the tangent modulus as function of stress for the intact esophagus and after separation into the muscle and mucosa-submucosa layers

Cross-sectional views of the esophagus and its two layers were obtained at the no-load state and zero-stress state. Upon reducing the no-load state to the zero-stress state by cutting the ring radially, the opened ring expanded itself into a sector with an opening angle of about 140° for the intact esophagus (figure 2, top). Separation of the mucosa-submucosa layer from the muscle layer resulted in the release of compressive forces in the mucosa-submucosal layer and tensile forces in the muscle layer. After separation the opening angle of the muscle and the mucosa-submucosa approached 45 and 90°, respectively. Statistically significant differences in opening angles were found between the intact segment and the mucosa-submucosa (p < 0.05), between the intact segment and the muscle layer (p < 0.05), and between the two separated layers (p < 0.05). In comparison to the specimens in the state of zero bending moment, the buckling of the mucosa became less apparent but still present after separation.

The stress-strain data are depicted in figure 2 (middle) in the sense of Kirchhoff stress and Green strain. A non-linear (exponential) curve was used to fit the data. The stress-strain curve for the submucosa-mucosa layer was located to the left of the curves for the muscle layer and for the intact esophagus at strains higher than 0.3, indicating that the submucosa-mucosa has the highest stiffness. Figure 2c shows the relationship between the tangent modulus and stress for the various layers. It can be seen that there is a linear relationship between the tangent modulus and the stress as a result of the exponential nature of the stress-strain relationship. Furthermore, the modulus is significantly higher in the submucosa layer than in the muscle layer and the intact esophagus as predicted from figure 2 (middle).

**Figure 3:**
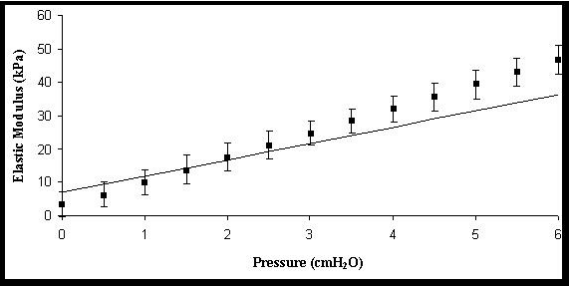
The elastic modulus as function of pressure for the intact esophagus. The curve with the error bar is the experimental date whereas the other curve is the theoretical curve. The theoretical fit is within one SD for the low pressure regime.

Figure 3 shows a composition of the predicted (Eq. 11) and measured Young's modulus for the intact esophagus. It can be seen that the agreement is good in the low stress-strain regime (pressure < 4 cmH_2_O) where the assumption of linear stress-strain relationship is most justified.

## Discussion

### Scope of Study and Major Findings

The peristaltic transport of swallowed material by the esophagus to the stomach is a neuromuscular function affected by a number of neuromuscular factors [9-14]. The nervous system and the contractile muscle behavior of the esophagus have been studied extensively [10,15] but the mechanics of the esophagus tissues is lagging far behind. The stress-strain-velocity history of the tissues of the esophagus is unknown. Since the esophagus is a tube, traditionally in mechanical analysis the wall material is treated as homogeneous without further analysis into layers. The effect of the two layers on the overall mechanics of the esophagus is examined in this article.

The unique structure of the esophagus as a composite of submucosa and muscle layers allows the separation of the two layers and an experimental determination of the constitutive properties of each layer. This is difficult to carry out in the blood vessels or in other organs. The coronary arteries can be separated at the external elastic laminae but only by tearing the adventitial layer [16]. The results show that the material properties differ between the esophageal intact wall, the muscle layer, and the submucosa layer. The submucosa layer is the stiffest.

### A Two-Layer Model

A major difference between the structures of the esophagus and blood vessels is the ease with which the wall can be separated into layers. This fact allowed us to obtain the stress-strain relation and the zero-stress state of the esophageal tissue layers, as reported above. In contrast, in spite of the extensive effort on theoretical and experimental bilayer models of arteries by Berry et al [17], Demiray and Vito [18], Maltzahn et al [19,20], and Rachev [21], the mechanical properties and the zero-stress states of the bi-layers of arteries are still largely unknown. For the esophagus, a practical question is: Can we regard the wall of the esophagus as a homogeneous tissue. Or must we treat it as composed of a mucosa-submucosa layer and a muscle layer? Or must we model it with even more detailed structures? Or can we model it as simply two concentric, non-interacting layers. The answer depends on the purpose of our investigation: What features of the organ does the investigator wish to know. In this article, we examined both the bi-layer model and the monolayer model, present a comparison and propose a simple model to explain the interaction of the two layers.

The opening angle of the inner submucosa layer is larger than that of the outer muscle layer which agrees with our previous report [1,3] and layered artery [22,23]. However the opening angles were largest in the intact layer and smallest in the muscle layer which differs somewhat from those obtained in guinea-pig [1] and in rabbit [3]. The difference may be species related but also due to experimental technique. In the previous studies esophageal rings were first cut radially and then separated circumferentially [1,3]. In the present study we first separated the inner submucosal tube from the outer muscle tube and then cut the ring radially in each layer. We believe that this procedure minimizes any damage inferred by the cutting. We plan to investigate the differences in experimental protocol to rule what causes the differences in opening angle between the previous studies and this study.

The stress-strain curve for the submucosa-mucosa layer was shifted to the left of the curves for the muscle layer and for the intact esophagus at strains higher than 0.3, indicating that the submucosa-mucosa has the highest stiffness (figure 2). This corresponds to the finding of a lower Green strain at pressure above 1.5 cmH2O (figure 1). The difference found below strain 0.3 and pressure 1.5 is due to that the submucosa-mucosa is compressed in the intact esophagus. Hence, when we study this layer after separation, it has a higher strain at low loads when compared to the other specimens. Furthermore, the submucosa-mucosa layer is rich in collagen. Thus, a contributing factor to the observed difference between layers may be that collagen during stretch first uncrimps with little resistance, then at higher loads has a high stiffness [7]. We observed that the stress-strain relationship of the intact esophagus and its two layers is exponential. The tangent modulus, which is the slope of the stress-strain relationship, varies exponentially with the strain (according to the stress-strain figure) and linearly with the stress. Hence, it is simpler to examine the modulus as function of stress. For a nonlinear stress-strain relationship it is meaningless to specify the modulus unless a stress or strain level is prescribed. Fung proposed that the slope of the tangent modulus-stress relationship, *α*, can be used as a measure of stiffness [24]. The data in this study clearly shows that the mucosa-submucosa layer is the stiffest which is in accordance with previous experience and the fact that submucosa contains large amounts of collagen. Therefore, the esophageal wall should be modelled as at least a two-layered composite system, as has also been proposed for arteries [18-20,22,23,25,26].

### Limitations of Study

A limitation of the study is that only uni-axial data were obtained in this study. Intuitively the circumferential direction seems to be the most important for cylindrical organs. Since longitudinal changes may also be important for esophageal function, future studies should implement bi- or tri-axial data. Another limitation is that the analytical model is restricted to the linear stress-strain regime. Furthermore, the esophagus and its layers are assumed to be cylindrical tubes, and that the esophageal tissue is incompressible. The last assumption is possibly true in the pressure range studied and it is also known from yet unpublished studies that the esophagus attains circular geometry both at the inner and outer surfaces even at low pressures. The linearity assumption needs to be generalized. We also assumed that each layer was homogeneous, though it is well known that the muscle layer is composed of longitudinal and circumferential muscle bundles. Hence, the muscle layer can be modeled into further sublayers.

## Conclusions and significance of research

We have developed an analytical tool that can be used to analyze the mechanics of bilayered organs. The model was used to study the esophagus. The model may be useful for studying the mechanical properties of other organs that can be separated into layers. There are two immediate implications of the results in this study for the understanding of esophageal function and for clinical practice. It is well known that pain may arise from the esophagus and that the receptors involved in the mechanotransduction are located at different positions in the wall. Detailed information about the stress and strain distributions in the layers is therefore important for the interpretation of receptor-mediated responses. Furthermore, the stress reduction during loading (caused by the residual stresses in the layers) may serve as a mechanism to reduce damage to the esophagus during excessive loadings caused by swallowing of large objects or by acute esophageal obstruction.

For the esophagus the model may be applied to the study of remodeling of the individual layers in response to disease. For example, in systemic sclerosis the muscle layers in esophagus are slowly replaced by fibrotic tissue, creating a passive conduit (fall pipe). It is already known that the esophageal stiffness increase in patients with systemic sclerosis [27,28] but we know very little about the mechanical remodeling in each layer.

## Authors contributions

Yanhua Fan carried out the experimental work, the measurements, and read and approved the final manuscript. Hans Gregersen designed the study, partly analyzed the data, and drafted the manuscript. Ghassan S. Kassab suggested the analysis, analyzed the data, drafted and approved the final manuscript.
